# Clinical characteristics and prognostic implications of diabetes and myocardial injury in patients admitted to the emergency room

**DOI:** 10.1186/s12872-021-02220-1

**Published:** 2021-08-30

**Authors:** Gil Bonet, Anna Carrasquer, Óscar M. Peiró, Raul Sanchez-Gimenez, Nisha Lal-Trehan, Victor del-Moral-Ronda, Isabel Fort-Gallifa, Alfredo Bardají

**Affiliations:** 1 Department of Cardiology, Joan XXIII University Hospital, Calle Dr Mallafré Guash 4, 43005 Tarragona, Spain; 2 Pere Virgili Health Research Institute (IISPV), Tarragona, Spain; 3 Rovira I Virgili University, Tarragona, Spain; 4 Clinical Laboratory, Catalan Institute of Health, Camp de Tarragona-Terres de L’Ebre, Tarragona, Spain

**Keywords:** Diabetes, Cardiac troponin, Myocardial injury, Mortality

## Abstract

**Background:**

This study aimed to investigate the clinical features and prognosis of diabetes and myocardial injury in patients admitted to the emergency department.

**Methods:**

We analyzed the clinical data of all consecutive patients admitted to the emergency department during the years 2012 and 2013 with at least 1 cardiac Troponin I (cTnI Ultra Siemens, Advia Centaur) determination, and were classified according to the status of diabetes mellitus (DM) and myocardial injury (MI). Clinical events were evaluated in a 4-year follow-up.

**Results:**

A total of 3622 patients were classified according to the presence of DM (n = 924 (25.55%)) and MI (n = 1049 (28.96%)). The proportion of MI in patients with DM was 40% and 25% in patients without DM. Mortality during follow-up was 10.9% in non-DM patients without MI, 21.3% in DM patients without MI, 40.1% in non-DM patients with MI, and 52.8% in DM patients with MI. A competitive risk model was used to obtain the Hazard Ratio (HR) for readmission for myocardial infarction or heart failure. There was a similar proportion of readmission for myocardial infarction and heart failure at a four-year follow-up in patients with DM or MI, which was much higher when DM was associated with MI, with respect to patients without DM or MI. The HR (95% Coefficient Interval) for myocardial infarction in the DM without MI, non-DM with MI, and DM with MI groups with respect to the non-DM without MI group was 2511 (1592–3960), 2682 (1739–4138), and 5036 (3221–7876), respectively. The HR (95% CI) for the risk of readmission for heart failure in the DM without MI, non-DM with MI, and DM with MI groups with respect to the non-DM without MI group was 2663 (1825–3886), 2562 (1753–3744) and 4292 (2936–6274), respectively.

**Conclusions:**

The association of DM and MI in patients treated in an Emergency Service identifies patients at very high risk of mortality and cardiovascular events.

**Supplementary Information:**

The online version contains supplementary material available at 10.1186/s12872-021-02220-1.

## Introduction

Diabetes mellitus (DM) is an important cardiovascular risk factor, and it is frequent among patients attending the emergency department with suspected acute coronary syndrome [1]. DM affects the prognosis of these patients, regardless of ruling out an acute coronary syndrome [2].

In patients with symptoms compatible with an acute coronary syndrome, cardiac troponin (cTn) determination can confirm or rule out myocardial infarction. cTn levels above the 99th percentile are considered myocardial injury (MI). Myocardial infarction is defined by pathology as myocardial cell death due to prolonged ischaemia. Type 1 myocardial infarction (T1MI), or spontaneous myocardial infarction related to ischaemia, is due to a primary coronary event such as plaque erosion and/or rupture, fissuring, or dissection. Type 2 myocardial infarction (T2MI), or secondary to ischaemia, is due to either increased oxygen demand or decreased supply [3]. Whatever the reason for the myocardial injury, the prognosis of these patients is adverse, whether they have a type 1 myocardial infarction [1], type 2 myocardial infarction [4], an acute or chronic non-ischemic myocardial injury [5].

In stable chronic patients with diabetes, it is well established that any elevation of cTn confers an adverse prognosis [6–14]. However, there is very little information on patients with diabetes treated in an emergency department in whom MI is detected [15, 16]. Therefore, the relative impact that diabetes has regarding myocardial injury or how both conditions are enhanced with the prognosis remains to be investigated. This study aims to acknowledge the clinical characteristics and prognostic implications of diabetes and myocardial injury in patients with a determination of cTn, treated in an emergency department.

## Methods

### Study population

This is a retrospective cohort study concerning all patients admitted to the university hospital’s emergency department between January 1, 2012 and December 31, 2013 that underwent at least one cTnI determination. Patients were identified using laboratory records. cTnI tests were performed according to the chest pain protocol of our center, although these biochemical analyses were also requested in patients with atypical symptoms or suspected acute coronary syndrome (ACS). For patients with more than one cTnI test, we selected the highest cTnI value. For those who were admitted to the emergency room several times, we included the first admission episode. The exclusion criteria were: (a) age under 18 years, (b) patients recovered from cardiac arrest, and (c) patients living outside our reference area. The local ethics committee approved the study.

### Cardiac Troponin I

All measurements of TnI were performed in the same laboratory using the contemporary immunoassay technique (TnI-Ultra from Siemens, Advia Centaur). According to the manufacturers, the upper and lower detection limits were 0.006 μg/ml and > 50 μg/ml. respectively. Levels below the detection limit were given a value of 0 and those above 50 μg/ml a value of 50. The reference range for a positive cTnI test was > 0.039 μg/ml, corresponding to the 99th percentile of a reference control group, with a coefficient of variation < 10%.

### Categorization of the study population

Diabetes status was defined based on the patient’s self-reported diagnosis or the use of anti-diabetic medications. MI was considered at any cTnI level above the reference 99th percentile.

### Clinical variables studied

Electronic medical records of all patients were reviewed. The demographic variables, cardiovascular risk factors, relevant cardiovascular and non-cardiovascular history, physical examination at the initial emergency evaluation, electrocardiographic findings, and laboratory tests were included. Glomerular filtration rate was calculated using the formula MDRD-4 (Diet modification in kidney disease). The primary diagnoses at discharge were also recorded.

Detection of an elevated cTn value above the 99th percentile upper reference limit (URL) was defined as myocardial injury (MI) [3]. The term myocardial infarction was applied to patients with both acute MI (i.e. elevated concentration of cardiac troponin [cTn] above the 99th percentile URL) and with concurrent acute myocardial ischaemia, whereas the term non ischaemic myocardial injury (NIMI) was applied in those with acute myocardial injury without ischaemia. Type 1 MI (T1MI) is caused by an acute atherothrombotic coronary event while type 2 MI (T2MI), also known as secondary MI, is a more heterogeneous entity, where an underlying condition other than acute atherothrombotic coronary artery disease contributes to an imbalance between myocardial oxygen supply and demand [3]. T1MI, T2MI and NIMI were defined by a consensus of two cardiologists, as previously reported [4], according to the criteria previously proposed by Saaby et al. [17].

### Primary and secondary endpoints

The primary outcome of the study was all-cause mortality at the 4-year follow-up. Secondary outcomes were readmission rates for heart failure or myocardial infarction. The combined event of death or readmission for myocardial infarction, or readmission for heart failure was considered for major cardiovascular events (MACE). The events in the follow-up were obtained from the electronic medical records of the patients and the death records.

### Statistical analysis

The baseline characteristics of the patients in the four categories were compared using the Kruskal–Wallis test for continuous variables and Pearson’s Chi^2^ test for categorical variables. Data are presented as medians and IQRs for continuous variables and as counts with percentages for categorical variables. Cox proportional hazards regression analysis was used in the univariate and multivariate mortality analysis. The variables included in the multivariate analysis were age and sex, cardiovascular risk factors (hypertension and smoking), relevant cardiovascular history (myocardial infarction, heart failure, peripheral arterial disease, and cerebrovascular disease) and variables related to co-morbidity (dementia, chronic obstructive pulmonary disease, and chronic kidney disease). The proportional hazard assumption was assessed by evaluating the constancy of the parallel lines drawn on the log–log plot and the Schoenfeld residuals. Death from any cause can be considered a competitive readmission event for heart failure and ACS. For this reason, a competing risks model was used to obtain readmission Hazard Ratio (HR) for heart failure and myocardial infarction. Cumulative incidence curves were drawn using the competing risks model. As discharge diagnoses can be a potential counfunder we performed an additional multivariate analyses for total mortality composed of age, atrial fibrillation and discharge diagnostics (heart failure, renal failure, anemia, cancer, respiratory pathology, sepsis and other infections)**.** Differences were considered statistically significant at *p* < 0.05. STATA V.13.0 (College Station, Texas, USA) was used for all analyzes.

## Results

### Baseline characteristics

**Fig. 1:**
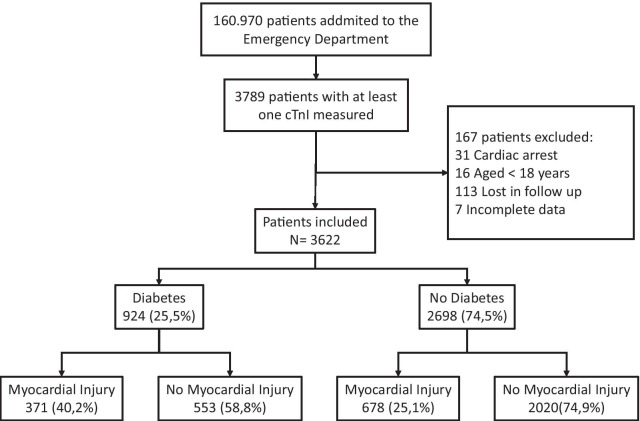
Flow diagram of patients. The distribution of patients in the four groups of the study is depicted. cTnI: Cardiac Troponin I

The total population included in the study was 3622 patients, who were classified according to the presence of DM (n = 924 (25.55%)) and MI (n = 1049 (28.96%)). The proportion of MI in patients with DM was 40.2% and 25.1% in patients without DM (Fig. 1, Additional file 1: Table S1). Thus, the population was distributed into four groups: patients without DM and without MI (n = 2020), patients with DM and without MI (n = 553, patients without DM and with MI, n = 678, and patients with DM and MI, n = 371. The demographic data, risk factors, cardiovascular and non-cardiovascular history, main symptoms on arrival at the emergency room, vital signs, ECG, and laboratory data in the four groups analyzed are described in Table 1.

**Table 1:** Clinical characteristics of the four groups of patients according to the status of diabetes and myocardial injury

	Total	DM		*p*	No DM		*p*
		MI	No MI		MI	No MI	
	3622	371	553		678	2020	
Age, years	68 (55–79)	76 (66–82)	73.5 (64–80)	< 0.001	83 (74–88)	77 (64–84)	< 0.001
Male sex	2068 (57.1)	218 (58.8)	296 (53.5)	0.116	430 (63.4)	1124 (55.6)	0.000
*Risk factors*							
Arterial Hypertension	2192 (60.5)	321 (86.5)	436 (78.8)	0.003	450 (66.4)	985 (48.8)	< 0.001
Current or previous smoker	1215 (33.5)	132 (35.6)	161 (29.1)	0.038	295 (43.5)	627 (31)	< 0.001
*Clinical history and comorbidities*							
Prior myocardial infarction	718 (19.8)	148 (39.9)	144 (26.0)	< 0.001	133 (19.6)	293 (14.5)	0.002
Congestive heart failure	257 (7.1)	60 (16.2)	43 (7.8)	< 0.001	74 (10.9)	80 (4.0)	< 0.001
Peripheral arterial disease	242 (6.7)	63 (17.0)	40 (7.2)	< 0.001	68 (10.0)	71 (3.5)	< 0.001
Stroke or TIA	248 (7.8)	59 (15.9)	46 (8.3)	< 0.001	74 (10.9)	105 (5.2)	< 0.001
Dementia	128 (3.5)	19 (5.1)	23 (4.2)	0.491	33 (4.9)	53 (2.6)	0.004
COPD	651 (18.0)	91 (24.5)	124 (22.4)	0.158	142 (20.9)	294 (14.6)	< 0.001
Mild liver disease	68 (1.9)	7 (1.9)	15 (2.7)	0.420	9 (1.3)	37 (1.8)	0.380
Moderate or severe liver disease	41 (1.1)	6 (1.6)	8 (1.4)	0.835	12 (1.8)	15 (0.7)	0.020
Renal disease	295 (8.1)	102 (27.5)	53 (9.6)	< 0.001	89 (13.1)	51 (2.5)	< 0.001
Cancer	395 (10.9)	45 (12.1)	73 (13.2)	0.632	93 (13.7)	184 (9.1)	0.001
Charlson index	4 (2–5)	6 (5–8)	5 (4–6)	< 0.001	4 (2–6)	3 (1–4)	< 0.001
*Symptoms*							
Chest pain	1891 (52.2)	168 (45.3)	264 (47.7)	0.463	347 (51.2)	1112 (55.0)	0.080
Dyspnea	605 (16.7)	117 (31.5)	84 (15.2)	< 0.001	164 (24.2)	240 (11.9)	< 0.001
Syncope	245 (6.8)	27 (7.3)	42 (7.6)	0.857	36 (5.3)	140 (6.9)	0.139
Other symptoms	1205 (33.3)	94 (25.3)	220 (39.8)	< 0.001	185 (27.3)	706 (45.0)	< 0.001
*Exploration [median]*							
HR (bpm)	79 (67–95)	86 (69–104)	80 (68–93)	< 0.001	105 (83–126)	90 (76–110)	< 0.001
SBP (mmHg)	138 (121–154)	140 (121–160)	140 (123–156)	0.792	156 (135–175)	152 (137–169)	0.045
Sat O	98 (96–100)	97 (94–99)	98 (96–99)	< 0.001	99 (97–100)	100 (99–100)	< 0.001
*Electrocardiogram*							
IVCD	528 (14.5)	92 (25.8)	84 (16.2)	0.001	131 (20.2)	221 (11.6)	< 0.001
Sinus rhythm	2780 (81.2)	259 (72.6)	423 (81.7)	0.001	457 (70.7)	1641 (86.2)	< 0.001
AF	573 (16.7)	82 (23.0)	87 (16.8)	0.023	170 (26.3)	234 (12.3)	< 0.001
Pacemaker stimulation	76 (2.2)	17 (4.8)	9 (1.7)	0.001	20 (3.1)	30 (1.6)	0.016
*Analytical tests [median]*							
Glucose (mg/dl)	111 (95–147)	180 (130–257)	151 (118–207)	< 0.001	151 (117–203)	118 (102–142)	< 0.001
Hemoglobin (g/dl)	13.4 (12.1–14.7)	12.4 (11–13.8)	13 (11.7–14.3)	< 0.001	14.7 (13.4–15.8)	14.9 (13.7–15.8)	< 0.001
Glomerular filtration rate	81 (60–100)	82 (55–105)	94 (76–115)	< 0.001	91 (68–115)	105 (86–123)	< 0.001

DM patients concerning non-DM patients were older and had more co-morbidities (hypertension, history of myocardial infarction and heart failure, peripheral vascular disease, cerebrovascular disease, chronic obstructive pulmonary disease, kidney disease, and history of neoplasms). Besides, they presented a worse Charlson index (Additional file 1: Table S1). **Patients with diabetes** showed less chest pain and more dyspnea, as the main symptom of consultation in the Emergency Department, worse oxygen saturation, and higher systolic blood pressure. In the ECG, they had a higher proportion of atrial fibrillation, and in the laboratory tests, lower hemoglobin and a worse glomerular filtration rate (Additional file 1: Table S1).

Hospital admission was more frequent among DM, and hospital mortality was significantly higher in people with DM than in non-DM (4.0% vs. 2.4%, *p* = 0.014) (Additional file 1: Table S3).

The differences between MI and non-MI in patients with and without DM are shown in Tables 1, 2, and 3. In both groups of patients, MI was associated with older age, more cardiovascular risk factors, more cardiovascular history and co-morbidity, an increased prevalence of atrial fibrillation in the ECG, and worse glomerular filtration and hemoglobin level.

**Table 2:** Principal diagnosis at the emergency department of the four groups of patients according to diabetes and myocardial injury status

	Total	DM		*p*	No DM		*p*
		MI	No MI		MI	No MI	
	3622	371	553		678	2020	
Acute Coronary Syndrome	439 (12.1)	131 (35.3)	22 (4.0)	< 0.001	246 (36.3)	40 (2.0)	< 0.001
Heart Failure	237 (6.5)	48 (17.8)	66 (8.7)	< 0.001	72 (10.6)	51 (2.5)	< 0.001
Tachyarrhythmia	219 (6.0)	16 (4.3)	25 (4.5)	0.880	56 (8.3)	122 (6.0)	0.044
Bradyarrhythmia	70 (1.7)	9 (2.4)	11 (2.0)	0.655	13 (1.9)	27 (1.3)	0.279
Hypertensive Crisis	52 (1.4)	2 (0.5)	8 (1.4)	0.191	4 (0.6)	38 (1.9)	0.019
Myocarditis	66 (1.8)	2 (0.5)	3 (0.5)	0.994	19 (2.8)	42 (2.1)	0.273
Syncope	197 (5.4)	12 (3.2)	41 (7.4)	0.007	14 (2.1)	130 (6.4)	< 0.001
Chest pain	957 (26.4)	12 (3.2)	156 (28.2)	< 0.001	22 (3.2)	767 (38.0)	< 0.001
Cerebrovascular disease	70 (1.9)	9 (2.4)	14 (2.5)	0.919	17 (2.5)	30 (1.5)	0.078
Respiratory Pathology	297 (8.2)	30 (8.1)	34 (6.1)	0.255	79 (11.7)	154 (7.6)	0.001
Pulmonary embolism	27 (0.3)	6 (1.6)	0	0.003	10 (1.5)	11 (0.5)	0.017
Gastrointestinal Pathology	286 (7.9)	14 (3.8)	61 (11.0)	< 0.001	17 (2.5)	194 (9.6)	< 0.001
Gastrointestinal bleeding	22 (0.6)	3 (0.8)	2 (0.4)	0.364	6 (0.9)	11 (0.5)	0.332
Renal Failure	23 (0.6)	9 (2.4)	3 (0.5)	0.013	9 (1.3)	2 (0.1)	< 0.001
Cancer	17 (0.5)	0	3 (0.5)	0.155	8 (1.2)	6 (0.3)	0.006
Anemia	37 (1.0)	4 (1.1)	9 (1.6)	0.487	6 (0.9)	18 (0.9)	0.988
Sepsis	22 (0.6)	7 (1.9)	3 (0.5)	0.053	9 (1.3)	3 (0.1)	< 0.001
Other Infections	53 (1.5)	9 (2.4)	10 (1.8)	0.517	10 (1.5)	24 (1.2)	0.562
Other diagnostics	527 (14.5)	30 (8.1)	99 (17.9)	< 0.001	54 (8.0)	344 (17.0)	< 0.001
T1MI	377 (10.4)	131 (35.5)			246 (36.3)		
T2MI	193 (5.3)	68 (18.3)			125 (18.4)		
NIMI	479 (13.2)	172 (47.4)			307 (45.3)		

Data represent the number (percentage)

*DM* diabetes mellitus, *MI* myocardial injury, *T1MI* Type 1 myocardial infarction, *T2MI* Type 2 myocardial infarction, *NIMI* non-ischemic myocardial infarction

**Table 3:** Clinical outcomes at 4-year follow-up of the four groups of patients according to diabetes and myocardial injury status

	Total	DM		*p*	No DM		*p*
		MI	No MI		MI	No MI	
	3622	371	553		678	2020	
*Hospitalization*							
Hospital admission	1183 (32.7)	247 (66.6)	127 (23.0)	< 0.001	487 (71.8)	322 (15.9)	< 0.001
In-hospital mortality	103 (2.8)	33 (8.9)	4 (0.7)	< 0.001	47 (6.9)	19 (0.9)	< 0.001
*4-year follow-up*							
Re-hospitalization for myocardial infarction	170 (4.7)	52 (14.0)	34 (6.1)	< 0.001	41 (6.0)	43 (2.1)	< 0.001
Re-hospitalization for heart failure	262 (7.2)	73 (19.7)	61 (11.0)	< 0.001	72 (10.6)	56 (2.8)	< 0.001
All-cause death	807 (22.3)	196 (52.8)	118 (21.3)	< 0.001	272 (40.1)	221 (10.9)	< 0.001

Data represent the number (percentage)

*DM* diabetes mellitus

### Events in the follow-up

**Fig. 2:**
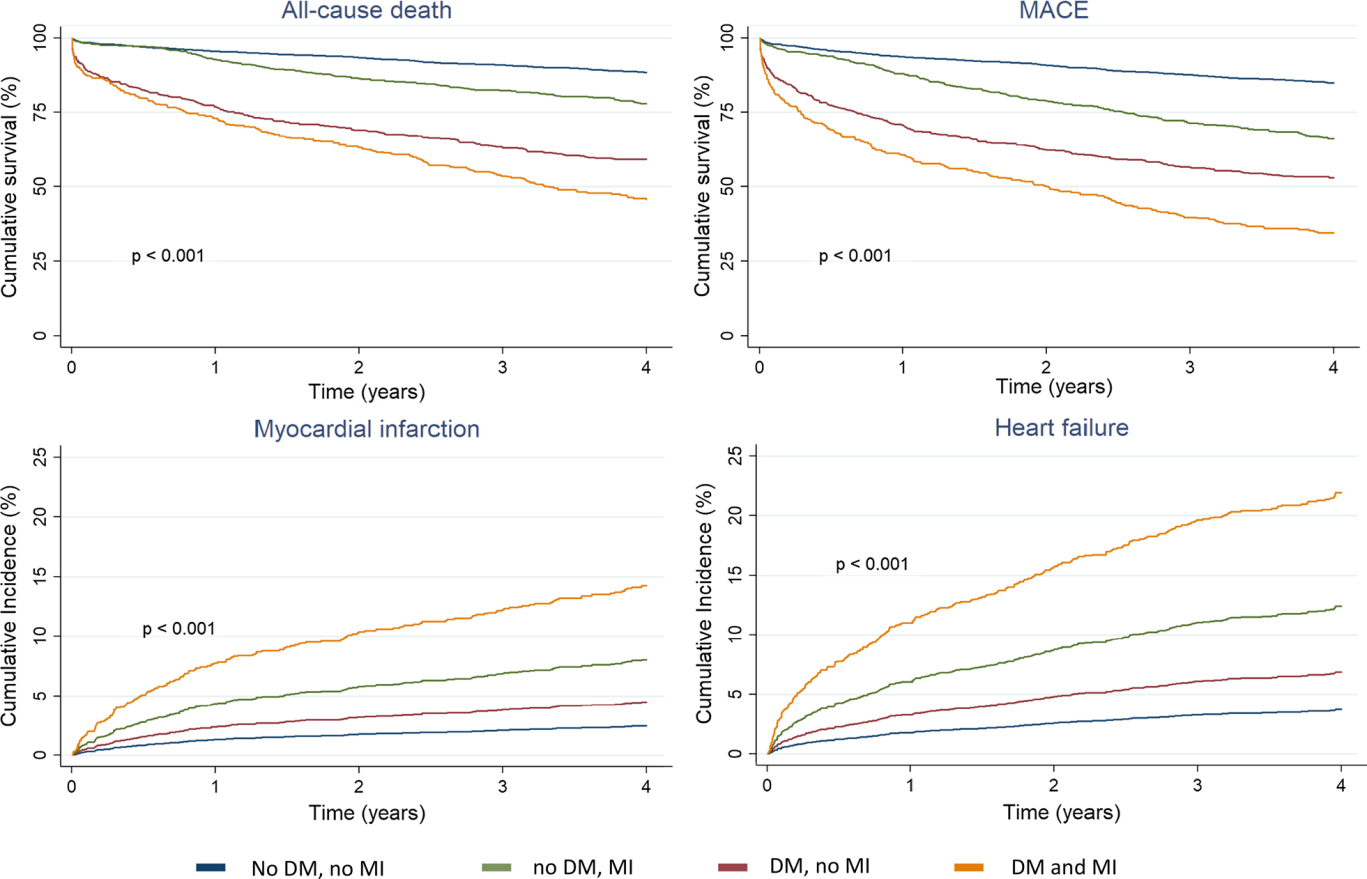
Adjusted survival and cumulative incidence in the four group of the study, for all-cause death, MACE (major adverse cardiovascular events), readmission for myocardial infarction and readmission for heart failure. DM: Diabetes mellitus. MI: Myocardial injury

Death during follow-up was 10.9% in non-DM patients without MI, 21.3% in DM patients without MI, 40.1% in non-DM patients with MI, and 52.8% in DM patients with MI (Fig. 2, Table 3). In the Additional file 1: Table S4, the univariate and multivariate model for predicting mortality for the different diagnostic groups is presented. Age, sex, history of heart failure, peripheral vascular disease, dementia, chronic obstructive pulmonary disease, and kidney disease were independent factors related to higher mortality. In the survival analysis at the four year follow-up, the four groups of patients had a significantly different trend with respect to non-DM patients without MI.

**Fig. 3:**
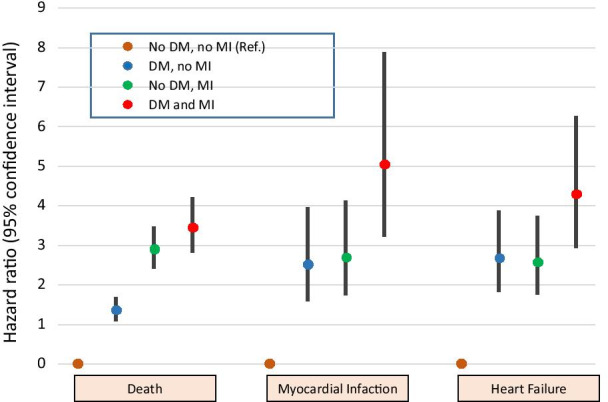
Forest plot showing adjusted hazard ratios for all-cause death, heart failure readmission and myocardial infarction readmission in the four groups of the study. DM: Diabetes mellitus. MI: Myocardial injury

A competing risks model was performed to obtain HR for readmission for myocardial infarction or heart failure. There was a similar proportion of readmission for myocardial infarction and heart failure at a four year follow-up in patients with DM or MI, and much higher when DM was associated with MI, with respect to patients without DM or MI. The HR (95% CI) for myocardial infarction in the DM without MI, non-DM with MI, and DM with MI groups with respect to the non-DM without MI group was 2,511 (1592–3960), 2682 (1739–4138), and 5036 (3221–7876), respectively (Fig. 3, Additional file 1: Table S4). The HR (95% CI) for the risk of readmission for heart failure in the DM without MI, non-DM with MI, and DM with MI groups with respect to the non-DM without MI group was 2663 (1825–3886), 2,562 (1753–3744) and 4,292 (2936–6274), respectively. (Fig. 3, Additional file 1: Table S5, S6).

## Discussion

Our study shows that MI detected in patients treated in an emergency department has a higher risk of mortality than DM in a 4-year follow-up and that the association of DM with MI increases this risk much further. On the other hand, DM is associated with a similar risk of myocardial infarction or heart failure with respect to MI. Again, the association of DM with MI further increases this risk.

Worldwide, cardiovascular disease (CVD) affects approximately one-third of patients with DM. CVD is one of the leading causes of mortality among people with DM and accounts for roughly half of all deaths in a 10-year follow-up [2]. In the prospective SMART cohort (Second Manifestations of ARTerial disease), patients with diabetes with CVD had a fourfold higher incidence of cardiovascular events and an eightfold higher incidence rate of vascular interventions compared to high-risk patients without DM2 and cardiovascular disease after adjusting for confounding factors. The incidence of the combination of non-fatal MI, non-fatal stroke, and vascular death was 5.8 per 1000 person-years in patients without DM2 or Cardiovascular disease at baseline and 40.7 per 1000 person-years in patients with DM2 and Cardiovascular disease at the beginning of the study [18].

The high prevalence of cTn levels above the 99th percentile of reference in the diabetic population is known to be associated with the presence of other traditional cardiovascular risk factors, such as age, sex, and kidney function [7]. Segre et al. studied the concentration of cTnI in patients with DM with and without underlying coronary artery disease [19]; they obtained a statistically significant elevation of cTnI in the group with underlying coronary artery disease compared to the group without coronary artery disease. The authors conclude that increased cTn concentrations are correlated with coronary heart disease in patients with diabetes. However, there are multiple cardiovascular risk factors associated with the presence of elevated Tnc in patients with diabetes. A sub-study of The Women’s Health Study makes it possible to estimate the prognostic importance of Tnc in diabetic women compared to non-diabetic women [11]. High-sensitivity cTnT was detectable in 45.5% of diabetic women and 30.3% of non-diabetic women (*p* = 0.0001). In models adjusted for traditional risk factors and hemoglobin A1c, detectable levels of high-sensitivity cTnT were associated with cardiovascular disease at follow-up (myocardial infarction, stroke, cardiovascular death) in diabetic individuals. Similarly, in the study by Yiu et al. in patients with diabetes, an elevated hs-TnI was associated with the combined event (MACE) of heart failure, myocardial infarction, and mortality at the 4-year follow-up [12]. Although multivariate analysis revealed that an elevated hs-TnI independently predicted MACE, the sensitivity (62.7%) and the positive predictive value (38.5%) were relatively low. However, a normal level of hs-TnI had an excellent negative predictive value (92.2%) for future MACE in patients with diabetes. Even the determination of Tnc in urine has been predictive of cardiovascular events in patients with diabetes. In the series by Chen, significantly higher levels of hs-TnI were observed in urine in those with subsequent incident CV events than in those without [20]. In studies with long-term follow-up, the association between Tnc levels and cardiovascular events in patients with diabetes is also observed.

In the ARIC (Atherosclerosis Risk in Com-munities) registry, cTn at the beginning of the study was strongly associated with mortality risk in a 10-year follow-up [14]. In the sub-analysis of the SAVOR-TIMI-53 (The Saxagliptin Assessment of Vascular Out-comes Recorded in Patients with Diabetes Mellitus (SAVOR) Thrombolysis in Myocardial Infarction (TIMI) 53 trial) study, in patients with diabetes with a single risk factor but no established CVD, elevated hs-cTnT identified people at high risk for cardiovascular death, hospitalization for heart failure, or myocardial infarction during a 2-year follow-up [6]. Hendriks et al. evaluated the association between hs-cTnT and mortality in patients with DM2 stratifying the study population according to hs-cTnT levels [21]. The authors found that hs-cTnT was associated with both cardiovascular mortality and all-cause mortality in a model adjusted for the main confounding factors. All of these studies in stable chronic patients agree with that we have observed in acute patients seen in the emergency room.

Some data indicate that the degree of DM control is also related to MI. Thus, the ARIC study (Risk of atherosclerosis in communities) examined the association between glycated hemoglobin (HbA1c) and high sensitivity cTnT [22]. Higher baseline HbA1c values were associated in a stepwise fashion with elevated cTnT (*p* for trend = 0.001). Therefore, there seems to be a relationship between HbA1c and serum cTn, and this, with cardiovascular events [23]. One of the additional mechanisms could be due to the effect of hyperglycemia on the reduction of glomerular filtration and the consequent decrease in cTn elimination, which also contributes to the elevation of cTn concentration.

Not only is the presence of elevated Tnc at baseline in patients with diabetes essential in evaluating their prognosis, but in population studies with long-term follow-up, the baseline presence of DM is associated with elevated Tnc during follow-up [8]. In the EXAMINE (Examination of Cardiovascular Outcomes With Alogliptin Versus Standard of Care) trial, serial evaluation of hsTnI revealed that a substantial proportion of patients with type 2 diabetes mellitus had persistently or dynamic values, and these were at high risk of recurrent episodes [10].

In fundamental studies, high blood glucose concentrations have been reported to cause MI through microcirculation dysfunction, increased oxidative stress, or other pathways [24, 25]. In patients with type 2 diabetes mellitus, hs-cTnT correlates with levels of the advanced glycation end-products in the skin, blood levels of brain natriuretic peptide, and reactive oxygen metabolites as markers of oxidative stress [9].

In the BARI 2D (Bypass Angioplasty Revascularization Investigation 2 Diabetes) study, baseline cTnT concentration in patients with diabetes with stable ischemic heart disease was abnormal in 39.3% of the patients [13]. The 5-year rate for the pooled endpoint was 27.1% among patients with abnormal baseline cTnT compared to 12.9% among those with normal baseline cTnT levels. In models that were adjusted for cardiovascular risk factors, diabetes severity, electrocardiographic abnormalities, and coronary anatomy, the HR for the pooled endpoint among patients with abnormal cTnT concentrations was 1.85, which is statistically significant. These data agree with our results: patients with diabetes seen in the emergency department in whom myocardial injury is detected have an increased risk of myocardial infarction during follow-up.

### Therapeutic implications

These findings have several implications. The presence of DM in patients seen in the emergency room should be an excellent opportunity to implement therapeutic measures, which have shown a decrease in cardiovascular events, in particular new admissions for heart failure and mortality [26]. It is not known whether these measures could be helpful in non-DM patients with MI, similar to the benefit that, for example, sodium-glucose co-transporter type 2 inhibitor (iSGLT2) has in the prevention of new admissions for non-DM heart failure patients. However, in the highest risk group, such as DM patients with MI, there is evidence of the potential cost-effectiveness of intensive diabetes treatment [27]. Therefore, it is now recognized that heart failure is one of the earliest manifestations of cardiovascular disease in patients with type 2 diabetes [28]; the determination of cTn could be helpful to identify patients at maximum risk. However, current treatment recommendations do not incorporate these biomarkers [29].

Another aspect to remark upon is the prevention of myocardial infarction. In the BARI 2D study, an abnormal cTnT value has not identified a subgroup of patients who benefited from randomization to accelerate coronary revascularization [13]. Therefore, it is doubtful whether the early identification of coronary disease through cardiac catheterization or an imaging technique with the aim of revascularization in these patients could be useful, beyond the implementation of all prevention measures.

### Limitations

Our study has several limitations. First, it is a broad series but in a single-center, so our conclusions can only serve as a working hypothesis which needs to be corroborated in other series. Second, we do not have accurate information on the long-term treatment that our patients received. Although this is an aspect left to the discretion of the treating physician or the primary care physician, specific cardiovascular prevention measures may have been underused. Third, we have analyzed MI in its entire spectrum of diagnostic possibilities, that is, type 1, type 2 myocardial infarction, and non-ischemic MI. And finallly, the main limitation of the work is the retrospective nature with possible selection bias. Diabetes status was defined based on the patient's self-reported diagnosis or use of anti-diabetic medications. Thus, many patients with undiagnosed DM could be misclassified**.**

### Conclusions

Our study reveals that the association between DM and MI in patients treated in an emergency department identifies patients at a very high risk of mortality and cardiovascular events. These patients are frequent and can represent an excellent opportunity to implement all available therapeutic measures to reduce their cardiovascular risk.

## Supplementary Information

**Additional file 1**. Supplemental Tables.

## Data Availability

Data available on request from the authors.

## Abbreviations

DM: Diabetes mellitus
MI: Myocardial injury
cTn: Cardiac troponin
cTnI: Cardiac Troponin I
cTnT: Cardiac Troponin T
HR: Hazard ratio
CI: Coefficient interval
CVD: Cardiovascular disease
