# Supplementary material for: Clinical characteristics and prognostic implications of diabetes and myocardial injury in patients admitted to the emergency room
Source: BMC Cardiovasc Disord. 2021 Aug 30;21:414. doi: 10.1186/s12872-021-02220-1 (PMC8404360; doi:10.1186/s12872-021-02220-1)
Supplement: Supplementary file 1 — Additional file 1. Supplemental Tables. [file 12872_2021_2220_MOESM1_ESM.docx]

**Supplemental material.**

Table 1S. Clinical characteristics of the four groups of patients according to the status of diabetes mellitus.

|  | Total | DM | no DM | p |
| --- | --- | --- | --- | --- |
|  | 3622 | 924 | 2698 |  |
| Age, years | 68(55-79) | 74(65-81) | 67(53-78) | <0.001 |
| Male sex | 2068(57.1) | 514(55.6) | 1554(57.6) | 0.296 |
| **Risk factors** |  |  |  |  |
| Arterial Hypertension | 2192(60.5) | 757(81.9) | 1435(53.2) | <0.001 |
| Current or previous smoker | 1215(33.5) | 293(31.7) | 922(34.2) | 0.171 |
| **Clinical History and comorbidities** |  |  |  |  |
| Prior myocardial infarction | 718(19.8) | 292(31.6) | 426(15.8) | <0.001 |
| Congestive heart failure | 257(7.1) | 103/11.1) | 154(5.7) | <0.001 |
| Peripheral arterial disease | 242(6.7) | 103(11.1) | 139(5.2) | <0.001 |
| Stroke or TIA | 248(7.8) | 105(11.4) | 179(6.6) | <0.001 |
| Dementia | 128(3.5) | 42(4.5) | 86(3.2) | 0.054 |
| COPD | 651(18.0) | 215(23.3) | 436(16.2) | <0.001 |
| Mild liver disease | 68(1.9) | 22(2.4) | 46(1.7) | 0.191 |
| Moderate or severe liver disease | 41(1.1) | 14/1.5) | 27(1.0) | 0.202 |
| Renal disease | 295(8.1) | 155(16.8) | 140(5.2) | <0.001 |
| Cancer | 395(10.9) | 118(12.8) | 277(10.3) | 0.035 |
| Charlson index, median (IQR) | 4(2-5) | 5(4-7) | 3(1-5) | <0.001 |
| **Symptoms** |  |  |  |  |
| Chest pain | 1891(52.2) | 432(46.8) | 1459(54.1) | <0.001 |
| Dyspnea | 605(16.7) | 201(21.8) | 404(15.0) | <0.001 |
| Syncope | 245(6.8) | 69(7.5) | 176(6.5) | 0.324 |
| Other symptoms | 1205(33.3) | 314(34.0) | 891(33.0) | 0.594 |
| **Exploration** |  |  |  |  |
| HR (bpm) | 79(67-95) | 82(68-98) | 78(66-93) | 0.091 |
| SBP (mmHg) | 138(121-154) | 140(122-158) | 137(121-153) | 0.002 |
| Sat O | 98(96-100) | 97(95-99) | 98(97-100) | <0.001 |
| **Electrocardiogram** |  |  |  |  |
| IVCD | 528(14.5) | 167(20.1) | 352(13.8) | <0.001 |
| Sinus rhythm | 2780(81.2) | 682(77.9) | 2098(82.3) | 0.005 |
| AF | 573(16.7) | 169(19.3) | 404(15.8) | 0.017 |
| Pacemaker stimulation | 76(2.2) | 26(3.0) | 50(2.0) | 0.080 |
| **Analytical tests** |  |  |  |  |
| Glucose (mg/dl) | 111(95-147) | 164(125-225) | 103(92-125) | <0.001 |
| Hemoglobin (g/dl) | 13.4(12.1-14.7) | 12.8(11.4-14.1) | 13.6(12.3-14.9) | <0.001 |
| Glomerular filtration rate | 81(60-100) | 68(45-99) | 82(62-102) | <0.001 |

Data represent the number (percentage) or median (interquartile range). Abbreviations. DM: Diabetes Mellitus. MI: Myocardial Injury. RIC: Interquartile range. TIA: Transit ischemic accident. COPD: Chronic obstructive pulmonary disease. SBP: Systolic blood pressure. HR: heart rate. Sat O: oxygen saturation. AF: Atrial Fibrillation. IVCD: Intra-ventricular conduction disorder

Table 2S. Principal diagnosis at the emergency department according to diabetes mellitus status.

|  | Total | DM | no DM | p |
| --- | --- | --- | --- | --- |
|  | 3622 | 924 | 2698 |  |
| Acute Coronary Syndrome | 439(12.1) | 153(16.6) | 286(10.6) | <0.001 |
| Heart Failure | 237(6.5) | 114(12.3) | 123(4.6) | <0.001 |
| Tachyarrhythmia | 219(6.0) | 41(4.4) | 178(6.6) | 0.017 |
| Bradyarrhythmia | 70(1.7) | 20(2.2) | 40(1.1) | 0.161 |
| Hypertensive Crisis | 52(1.4) | 10(1.1) | 42(1.6) | 0.295 |
| Myocarditis | 66(1.8) | 5(0.5) | 61(2.3) | 0.001 |
| Syncope | 197(5.4) | 53(5.7) | 144(5.3) | 0.645 |
| Chest pain | 957(26.4) | 168(18.2) | 789(29.2) | <0.001 |
| Cerebrovascular disease | 70(1.9) | 23(2.5) | 47(1.7) | 0.155 |
| Respiratory Pathology | 297(8.2) | 64(6.9) | 233(8.6) | 0.102 |
| Pulmonary embolism | 27(0.3) | 6(0.5) | 21(2.3) | 0.001 |
| Gastrointestinal Pathology | 286(7.9) | 75(8.1) | 211(8.6) | 0.773 |
| Gastrointestinal bleeding | 22(0.6) | 5(0.5) | 17(0.6) | 0.764 |
| Renal Failure | 23(0.6) | 12(1.3) | 11(0.4) | 0.003 |
| Cancer | 17(0.5) | 3(0.3) | 47(1.7) | 0.456 |
| Anemia | 37(1.0) | 13(1.4) | 24(0.9) | 0.177 |
| Sepsis | 22(0.6) | 10(1.1) | 12(0.4) | 0.031 |
| Other Infections | 53(1.5) | 19(2.1) | 34(1.3) | 0.082 |
| Other diagnostics | 527(14.5) | 129(14.0) | 398(14.8) | 0.556 |
|  |  |  |  |  |
| T1MI | 377(10.4) | 131(14.2) | 247(9.1) | <0.001 |
| T2MI | 193(5.3) | 68(7.4) | 125(4.6) | 0.001 |
| NIMI | 479(13.2) | 172(18.6) | 307(11.4) | <0.001 |

Data represent the number (percentage). Abbreviations. DM: Diabetes Mellitus. MI: Myocardial Injury. T1MI: Type 1 myocardial infarction. T2MI: Type 2 myocardial infarction. NIMI: non-ischemic myocardial infarction.

Table 3S. Clinical outcomes at 4-year follow-up of the four groups of patients according to diabetes status.

|  | Total | DM | no DM | p |
| --- | --- | --- | --- | --- |
|  | 3622 | 924 | 2698 |  |
| **Hospitalization** |  |  |  |  |
| Hospital admission | 1183(32.7) | 374(40.5) | 809(30.0) | <0.001 |
| In-hospital mortality | 103(2.8) | 37(4.0) | 66(2.4) | 0.014 |
| **4-year follow-up** |  |  |  |  |
| Re-hospitalization for myocardial infarction | 170(4.7) | 86(9.3) | 84(3.1) | <0.001 |
| Re-hospitalization for heart failure | 262(7.2) | 134(14.5) | 128(4.7) | <0.001 |
| All-cause death | 807(22.3) | 314(34.0) | 493(18.3) | <0.001 |

Data represent the number (percentage), Abbreviations. DM: Diabetes Mellitus.

Table 4S. Analysis using Cox regression for total death in the follow-up. Variables included in the raw (Univariate) model and variables in the adjusted model (Multivariate). MODEL 1

|  | Univariate | | | |  | Multivariate | | | |
| --- | --- | --- | --- | --- | --- | --- | --- | --- | --- |
|  | HR | 95.0% CI | |  |  | HR | 95.0% CI | |  |
|  |  | Inferior | Superior | p |  |  | Inferior | Superior | p |
|  |  |  |  |  |  |  |  |  |  |
| Age | 1.076 | 1.069 | 1.083 | <0.001 |  | 1.063 | 1.056 | 1.070 | <0.001 |
| Male Sex | 0.999 | 0.869 | 1.148 | 0.989 |  | 1.209 | 1.047 | 1.396 | 0.010 |
|  |  |  |  |  |  |  |  |  |  |
| Arterial Hypertension | 2.573 | 2.175 | 3.043 | <0.001 |  |  |  |  |  |
| Current or previous smoker | 1.063 | 0.920 | 1.228 | 0.410 |  |  |  |  |  |
|  |  |  |  |  |  |  |  |  |  |
| Prior myocardial infarction | 1.729 | 1.485 | 2.014 | <0.001 |  |  |  |  |  |
| Congestive heart failure | 3.400 | 2.831 | 4.083 | <0.001 |  | 1.616 | 1.337 | 1.953 | <0.001 |
| Peripheral arterial disease | 2.350 | 1.914 | 2.885 | <0.001 |  | 1.382 | 1.140 | 1.676 | 0.001 |
| Stroke or TIA | 2.543 | 2.104 | 3.074 | <0.001 |  |  |  |  |  |
|  |  |  |  |  |  |  |  |  |  |
| Dementia | 3.548 | 2.785 | 4.522 | <0.001 |  | 1.668 | 1.299 | 2.142 | <0.001 |
| COPD | 2.174 | 1.871 | 2.527 | <0.001 |  | 1.419 | 1.217 | 1.654 | <0.001 |
| Renal disease | 3.699 | 3.115 | 4.392 | <0.001 |  | 1.510 | 1.256 | 1.815 | <0.001 |
|  |  |  |  |  |  |  |  |  |  |
| No DM, no MI | Ref. |  |  |  |  |  |  |  |  |
| DM, no MI | 2.009 | 1.607 | 2.512 | <0.001 |  | 1.352 | 1.080 | 1.693 | 0.009 |
| no DM, MI | 4.546 | 3.806 | 5.429 | <0.001 |  | 2.896 | 2.413 | 3.477 | <0.001 |
| DM and MI | 6.440 | 5.312 | 7.809 | <0.001 |  | 3.441 | 2.809 | 4.216 | <0.001 |

Abbreviations. HR: Hazard Ratio. CI: coefficient interval. DM: Diabetes Mellitus. MI: Myocardial Injury. TIA: Transit ischemic accident. COPD: Chronic obstructive pulmonary disease.

Table 5S. Analysis using Cox regression for total death in the follow-up. Variables included in the raw (Univariate) model and variables in the adjusted model (Multivariate). MODEL 2

|  | Univariate | | | |  | Multivariate | | | |
| --- | --- | --- | --- | --- | --- | --- | --- | --- | --- |
|  | HR | 95.0% CI | |  |  | HR | 95.0% CI | |  |
|  |  | Inferior | Superior | p |  |  | Inferior | Superior | p |
|  |  |  |  |  |  |  |  |  |  |
| Age | 1.076 | 1.069 | 1.083 | <0.001 |  | 1.064 | 1.057 | 1.072 | <0.001 |
|  |  |  |  |  |  |  |  |  |  |
| Atrial fibrillation | 2.345 | 2.000 | 2.744 | <0.001 |  |  |  |  |  |
|  |  |  |  |  |  |  |  |  |  |
| Heart Failure | 3.265 | 2.699 | 3.949 | <0.001 |  | 1.702 | 1.380 | 2.099 | <0.001 |
| Renal Failure | 2.499 | 1.378 | 4.530 | 0.003 |  |  |  |  |  |
| Anemia | 2.506 | 1.571 | 3.999 | <0.001 |  | 2.365 | 1.453 | 3.850 | 0.001 |
| Cancer | 9.883 | 5.916 | 16.508 | <0.001 |  | 10.183 | 5.786 | 17.922 | <0.001 |
| Respiratory Pathology | 2.027 | 1.660 | 2.475 | <0.001 |  | 1.608 | 1.288 | 2.001 | <0.001 |
| Sepsis | 5.524 | 3.255 | 9.373 | <0.001 |  | 2.984 | 1.636 | 5.444 | <0.001 |
| Other Infections | 2.123 | 1.376 | 3.275 | 0.001 |  | 1.714 | 1.040 | 2.826 | 0.035 |
|  |  |  |  |  |  |  |  |  |  |
| No DM, no MI | Ref. |  |  |  |  |  |  |  |  |
| DM, no MI | 2.009 | 1.607 | 2.512 | <0.001 |  | 1.432 | 1.132 | 1.812 | 0.003 |
| no DM, MI | 4.546 | 3.806 | 5.429 | <0.001 |  | 2.925 | 2.410 | 3.550 | <0.001 |
| DM and MI | 6.440 | 5.312 | 7.809 | <0.001 |  | 4.071 | 3.316 | 4.998 | <0.001 |

Abbreviations. HR: Hazard Ratio. CI: coefficient interval. DM: Diabetes Mellitus. MI: Myocardial Injury.

Table 6S. Analysis using Cox regression for myocardial infarction in the follow-up. Variables included in the raw (Univariate) model and variables in the adjusted model (Multivariate).

|  | Univariate | | | |  | Multivariate | | | |
| --- | --- | --- | --- | --- | --- | --- | --- | --- | --- |
|  | HR | 95.0% CI | |  |  | HR | 95.0% CI | |  |
|  |  | Inferior | Superior | p |  |  | Inferior | Superior | p |
|  |  |  |  |  |  |  |  |  |  |
| Age | 1.059 | 1.049 | 1.069 | 0.001 |  | 1.038 | 1.025 | 1.051 | 0.001 |
| Male Sex | 0.668 | 0.524 | 0.851 | 0.001 |  | 0.748 | 0.576 | 0.973 | 0.03 |
|  |  |  |  |  |  |  |  |  |  |
| Arterial Hypertension | 3.63 | 2.601 | 5.065 | 0.001 |  | 1.534 | 1.064 | 2.209 | 0.022 |
| Current or previous smoker | 0.832 | 0.638 | 1.084 | 0.173 |  |  |  |  |  |
|  |  |  |  |  |  |  |  |  |  |
| Prior myocardial infarction | 1.875 | 1.444 | 2.435 | 0.001 |  |  |  |  |  |
| Congestive heart failure | 6.182 | 4.716 | 8.103 | 0.001 |  | 3.047 | 2.236 | 4.15 | 0.001 |
| Peripheral arterial disease | 2.397 | 1.694 | 3.39 | 0.001 |  |  |  |  |  |
| Stroke or TIA | 1.854 | 1.299 | 2.645 | 0.001 |  |  |  |  |  |
|  |  |  |  |  |  |  |  |  |  |
| Dementia | 0.905 | 0.447 | 1.833 | 0.781 |  | 0.458 | 0.219 | 0.954 | 0.038 |
| COPD | 3.075 | 2.398 | 3.943 | 0.001 |  | 2.144 | 1.636 | 2.81 | 0.001 |
| Renal disease | 2.989 | 2.206 | 4.05 | 0.001 |  |  |  |  |  |
|  |  |  |  |  |  |  |  |  |  |
| No DM, no MI |  |  |  |  |  |  |  |  |  |
| DM, no MI | 1.676 | 1.261 | 2.227 | 0.001 |  | 2.663 | 1.825 | 3.886 | 0.001 |
| no DM, MI | 1.732 | 1.319 | 2.274 | 0.001 |  | 2.562 | 1.753 | 3.744 | 0.001 |
| DM and MI | 3.722 | 2.839 | 4.881 | 0.001 |  | 4.292 | 2.936 | 6.274 | 0.001 |

Abbreviations. HR: Hazard Ratio. CI: coefficient interval. DM: Diabetes Mellitus. MI: Myocardial Injury. TIA: Transit ischemic accident. COPD: Chronic obstructive pulmonary disease.

Table 7S. Analysis using Cox regression for heart failure in the follow-up. Variables included in the raw (Univariate) model and variables in the adjusted model (Multivariate).

|  | Univariate | | | |  | Multivariate | | | |
| --- | --- | --- | --- | --- | --- | --- | --- | --- | --- |
|  | HR | 95.0% CI | |  |  | HR | 95.0% CI | |  |
|  |  | Inferior | Superior | p |  |  | Inferior | Superior | p |
|  |  |  |  |  |  |  |  |  |  |
| Age | 1.017 | 1.008 | 1.027 | 0.001 |  |  |  |  |  |
| Male Sex | 1.438 | 1.049 | 1.972 | 0.024 |  |  |  |  |  |
|  |  |  |  |  |  |  |  |  |  |
| Arterial Hypertension | 2.08 | 1.464 | 2.954 | 0.001 |  |  |  |  |  |
| Current or previous smoker | 1.433 | 1.056 | 1.943 | 0.021 |  |  |  |  |  |
|  |  |  |  |  |  |  |  |  |  |
| Prior myocardial infarction | 3.549 | 2.624 | 4.801 | 0.001 |  | 2.524 | 1.807 | 3.526 | 0.001 |
| Congestive heart failure | 1.374 | 0.821 | 2.3 | 0.226 |  |  |  |  |  |
| Peripheral arterial disease | 2.997 | 2.008 | 4.474 | 0.001 |  | 1.599 | 1.042 | 2.451 | 0.031 |
| Stroke or TIA | 1.586 | 0.993 | 2.534 | 0.053 |  |  |  |  |  |
|  |  |  |  |  |  |  |  |  |  |
| Dementia | 0.69 | 0.255 | 1.866 | 0.465 |  |  |  |  |  |
| COPD | 1.225 | 0.848 | 1.769 | 0.279 |  |  |  |  |  |
| Renal disease | 2.659 | 1.793 | 3.942 | 0.001 |  |  |  |  |  |
|  |  |  |  |  |  |  |  |  |  |
| No DM, no MI | Ref. |  |  |  |  |  |  |  |  |
| DM, no MI | 1.366 | 0.94 | 1.984 | 0.102 |  | 2.511 | 1.592 | 3.96 | 0.001 |
| no DM, MI | 1.425 | 1.002 | 2.024 | 0.048 |  | 2.682 | 1.739 | 4.138 | 0.001 |
| DM and MI | 4.184 | 3.013 | 5.809 | 0.001 |  | 5.036 | 3.221 | 7.876 | 0.001 |

Abbreviations. HR: Hazard Ratio. CI: coefficient interval. DM: Diabetes Mellitus. MI: Myocardial Injury. TIA: Transit ischemic accident. COPD: Chronic obstructive pulmonary disease.
